# Pinocembrin ameliorates arrhythmias in rats with chronic ischaemic heart failure

**DOI:** 10.1080/07853890.2021.1927168

**Published:** 2021-06-01

**Authors:** Yan Guo, Cui Zhang, Tianxin Ye, Xiuhuan Chen, Xin Liu, Xiaoli Chen, Yazhou Sun, Chuan Qu, Jinjun Liang, Shaobo Shi, Bo Yang

**Affiliations:** a Department of Cardiology, Renmin Hospital of Wuhan University, Wuhan, China; b Cardiovascular Research Institute, Wuhan University, Wuhan, China; c Hubei Key Laboratory of Cardiology, Wuhan, China

**Keywords:** Chronic ischaemic heart failure, ventricular arrhythmias, pinocembrin, autonomic remodelling, structural remodelling

## Abstract

**Objective:**

Ventricular arrhythmias (VAs) are a common complication of chronic ischaemic heart failure (CIHF). The purpose of this study is to investigate the efficacy of pinocembrin in a rat model of VAs induced by CIHF and further examine the possible mechanism.

**Methods:**

Rats were subjected to ligation of left anterior descending coronary artery to mimic CIHF and then received pinocembrin treatment daily for 2 months. The vivo electrophysiology were performed to determine the effect of pinocembrin on ventricular electrical activity. The expression of Cav1.2, Kv4.2, and NGF was determined by Western blot. The structural change of ventricle was tested by the Echocardiography, Masson staining, and HE staining. The effect of pinocembrin on sympathetic nerve-related markers was detected by the immunostaining and the ELISA was used to test for biomarkers associated with heart failure.

**Results:**

Pinocembrin increased the expression of ion channel protein Cav1.2 and Kv4.3, ameliorated the shortening of action potential duration (APD) and reduced the incidence and duration of ventricular fibrillation (VF). Pinocembrin also reduced the expression of nerve growth factor (NGF) and improved the autonomic nerve remodelling. In addition, pinocembrin reduced the area of infarct area and myocardial fibrosis, accompanied by increasing the expression of connexin protein 43 (C_X_43).

**Conclusion:**

We demonstrate that pinocembrin reduces cardiac nerve remodelling and protects against Vas induced by CIHF. The findings suggest that pinocembrin can be a promising candidate for the treatment of VAs.

## 1. Introduction

In recent decades, thanks to the reperfusion therapy and better treatment for post-infarction acute arrhythmias, survival rates have improved in patients with acute myocardial infarction (MI) [1]. However, the prevalence of ventricular arrhythmias (VAs) and consequent sudden cardiac death (SCD) from chronic ischaemic heart failure (CIHF) caused by MI is increasing significantly [2]. Both animal and clinical studies have shown that MI predispose patients to CIHF, of which degenerative heart condition is the main cause of SCD [3]. However, targeted therapy to alleviate arrhythmia susceptibility in CIHF patients remains a challenge. Unlike the arrhythmia of acute phase after MI, which is mainly due to the interference of resting membrane potential and electrical uncoupling consequently, the mechanism of arrhythmias in CIHF is not clear. One potential therapeutic mechanism is the blocking of neural remodelling. When heart failure manifests as systolic dysfunction, an adaptive sympathetic nervous system activation occurs in an attempt to maintain a normal cardiac output. And if this becomes excessive, the risk of developing VAs increases. For instance, stimulation of left stellate ganglion has a pro-arrhythmic effect [4]. Cardiac sympathetic denervation in animal models reduces the induction of ventricular tachyarrhythmias (VTs) and VAs due to CIHF [5]. On the contrary, chronic electrical stimulation of the vagus nerve enhances the ventricular fibrillation threshold in ambulatory dogs [6].

Pinocembrin, one of the flavonoids, mainly extracted from propolis [7]. Pinocembrin contain several active functional groups such as hydroxyl group [8], which has anti-inflammation and negative ischaemia/reperfusion (I/R) effects in cardiac tissues. It also alleviates the MI area [9], inhibits myocyte apoptosis [10], resists calcium overloading, and reduces the susceptibility to atrial fibrillation. On the other hand, pinocembrin improves neuronal degeneration in Alzheimer’s disease [11]. Moreover, in cerebral I/R, pinocembrin reduces infarct size, improves behavioural dysfunction, and attenuates neuronal damage [12].

Based on both neuroprotective and cardioprotective effects of pinocembrin, we hypothesize that the effects of pinocembrin on VAs induced by CIHF may be promising. However, pinocembrin efficacy in the treatment of CIHF-related VAs remains unknown. In this study, we examined the effects of pinocembrin treatment on VAs in rats of CIHF and explored the possible mechanisms.

## 2. Materials and methods

### 2.1. Animals

Sprague–Dawley male rats (200–250 g) purchased from Wuhan University Experimental Animal Center, China. Rats were housed in specific pathogen-free facilities conditions with free access to food and water. After approximately 5 days of adaptive feeding, 45 SD male rats were randomized into SHAM (*n* = 15), CIHF (*n* = 15), and CIHP (CIHF + pinocembrin, *n* = 15) groups. All animal experiments were approved by the Animal Experimental Administration of Wuhan University (Animal Ethical Number: SY2019-015) and were conducted in accordance with the *Guide for the Care and Use of Laboratory Animals*.

### 2.2. HF rat model

The model of CIHF reported here is based on the established rat model after MI. After being anaesthetized with pentobarbital sodium (3%, 0.3 ml/kg) and nalbuphine HCl (2 mg/kg), rats were ventilated with room air by a respirator (KW-100-2, Karwin Biotechnology Co., Nanjing, China). The rats in the CIHF and CIHP groups were subjected to a thoracotomy and ligation of left anterior descending coronary artery, which caused transmural MI. Monitoring ST segment elevation by two-lead ECG. At the same time, the rats in the SHAM group only had a thoracotomy. All rats were injected intramuscularly with penicillin (200,000 IU) twice daily for 1 week. After successful establishment of the MI model, follow-up procedures were carried out after 2 months of normal feeding.

### 2.3. Pinocembrin administration

Pinocembrin (purity > 99%), purchased from Sigma (St. Louis, MO), was dissolved in saline. Two months after the operation, animals in the CIHP group received pinocembrin (5 mg/kg) by tail vein daily for 2 months [13]. The other two groups received the same volume injection of saline.

### 2.4. Echocardiography

The rats were given echocardiography before and after the treatment with pinocembrin. Cardiac function was assessed using 2D and M-mode (VINNO 6, Vinno Corporation, Suzhou, China) in a standard long axis section of the left ventricle view.

### 2.5. *In vivo* electrophysiology

Animals were anaesthetized with pentobarbital sodium (3%,0.3 ml/kg), and then performed intubation and ventilation (KW-100-2, Karwin Biotechnology Co., Nanjing, China). Under deep anaesthesia, the rats were subjected to thoracotomy to expose the heart. Following the standard program, stimulation was performed using a custom-made stimulator. The recording electrode was placed at two epicardial sites: peri-infarct zone (-PZ) and remote zone (-RZ). Signals were recorded and analyzed using the PowerLab system (4/35, AD Instruments, Bella Vista, Australia) and LabChart 8.0 software.

#### 2.5.1. Electrocardiogram recording

Lead II of ECG was continuously monitored during the procedure. The RR interval and QT interval was measured during 5 min to calculate the plot-Poincare maps and QTc.

#### 2.5.2. Measurement of effective refractory period

Programmed stimulation (S1–S2) composing of eight consecutive stimuli (S1, 5 V, 2 ms pulse width, and 200 ms interval), followed by an extra stimulus (S2, 5 V, 2 ms pulse width, and decreasing from 100 ms by 2 ms) was delivered to measure the effective refractory period (ERP).

#### 2.5.3. Monophasic action potential recording

Action potential duration (APD) was measured using a dynamic steady-state pacing protocol (S1–S1) that consists of 10 consecutive stimuli (S1, 5 V, 2 ms pulse width). The initial pacing cycle length (PCL) was slightly shorter than the sinus cycle length.

#### 2.5.4. VFS inducibility

Ventricular fibrillations (VFs) were measured using a burst pacing (S1, 5 V, 0.2 ms pulse width, 2 ms internal, 2 s duration) repeated 20 times, with VFs defined as rapid irregular rhythm maintaining for at least 2 s.

### 2.6. Histological examination

#### 2.6.1. Immunostaining of LV tissue

LV samples were fixed with 4% paraformaldehyde, encased in paraffin, sliced into 6-µm sections and incubated with primary antibody to growth associated protein 43 (GAP_43_) (1:300, Abcam, Cambridge, UK), tyrosine hydroxylase (TH) (1:100, Abcam) or connexin 43 (C_X_43) (1:1000, Abcam) at 4 °C overnight. The tissues then incubated with peroxidase-coupled goat anti-rabbit or anti-mouse secondary antibodies (1:5000) for 1 h at room temperature. The mean density in the three fields with the highest nerve density was examined under 200× microscope. Nerve density was automatically measured by a quantitative digital image analysis system (Image Pro-Plus, version 6.0, Media Cybernetics Inc., Rockville, MD).

#### 2.6.2. Fibrosis and infarct area quantification

The paraffin sections of the ventricle were stained with Masson’s trichrome and HE, respectively. The collagen fibrils and infarct area were analyzed by Image-Pro Plus 6.0 software (SPSS Inc., Chicago, IL).

### 2.7. Western blot analysis

Ventricles were harvested quickly, washed with saline, and stored at −80 °C until the next step. Proteins of frozen tissues were lysed with lysis buffer (20 mM Tris-HCl, 1 mM Na_3_VO_4_, and 5 mM NaF), then separated by 8–12% SDS-PAGE and transferred to polyvinylidene difluoride (PVDF) membranes. After 5% skimmed milk blocking, PVDF membranes were incubated with primary antibodies overnight, such as Cav1.2 (1:1000; Abcam), NGF (1:200; Abcam), Kv4.2 (1:1000; Abcam), and GAPDH (1:10,000; Abcam), followed by incubation with appropriate secondary antibody (peroxidase-coupled goat anti-rabbit or goat anti-mouse, 1:1000, Abcam). Membranes were analyzed by an Image-Quant ECL Imager (GE Healthcare, Chicago, IL).

### 2.8. ELISA

Blood was extracted from the inferior vena cava for ELISA as described in the previous work [13]. NT-proBNP was detected. The procedure of ELISA was conducted in accordance with the manufacturers’ specification.

### 2.9. Statistical analysis

All data were expressed as means ± SEM. *p* < .05 was considered significant. Unpaired, two-tailed *t*-test were used for two groups and one-way ANOVA followed by Bonferroni’s multiple comparisons test for groups of three or more. All statistical tests were performed with GraphPad Prism v8 software (GraphPad Software, La Jolla, CA).

## 3. Results

### 3.1. Effect of pinocembrin on rat heart function after CIHF

**Figure 1.**
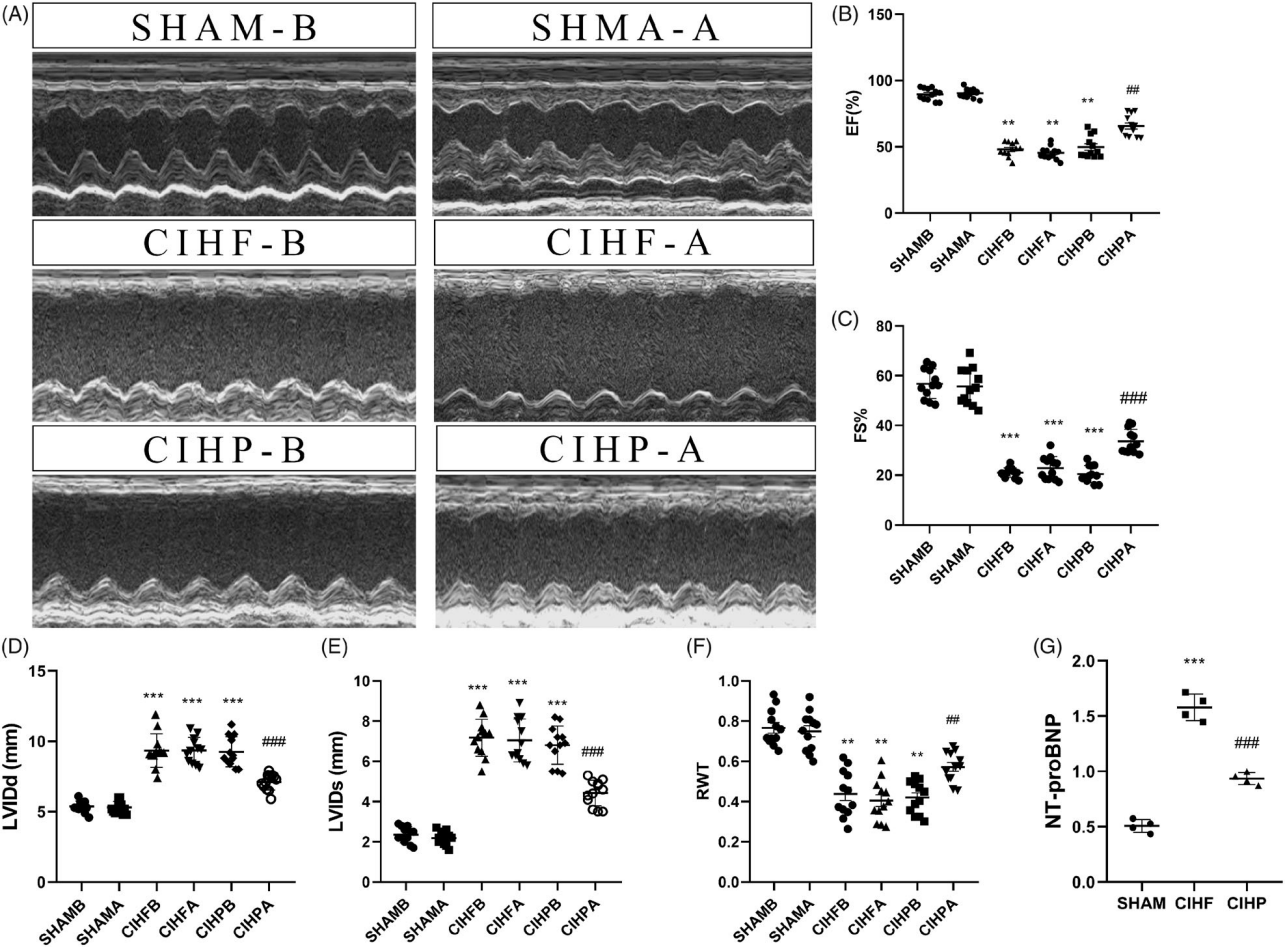
Assessment of cardiac function by echocardiogram(*n* = 15) and ELISA(*n* = 3) in each group. (A) Representative images of echocardiogram. (B–F) Statistical analysis of EF, FS, LVIDd, LVIDs and RWT in the three groups. (G) NT-proBNP concentrations of each group, respectively. Data are expressed as means ± SE. **p < .01 versus SHAM; ##*p* < .01 versus CIHF, ***p < .001 versus SHAM, ###*p* < .001 versus CIHF. (A) After the treatment with pinocembrin. (B) Before the treatment with pinocembrin, LVIDd: left ventricular end-diastolic dimension; LVIDs: left ventricular end-systolic dimension; LVEF: ejection fraction; FS: fractional shortening; RWT: relative wall thickness.

We detected cardiac function by echocardiography before and after the pinocembrin treatment (Figure 1(A)). Echocardiography before intervention revealed that the SHAM, CIHF, and CIHP group had an average ejection fraction (EF) of 89.74 ± 1.2%, 45.39 ± 1.32%, and 48.23 ± 1.44%, respectively (Figure 1(B)). As shown by echocardiography, the increased LVIDd and LVIDs and decreased FS were observed in the CIHF group (Figure 1(C–E)). The RWT of each group (SHAM, CIHF, and CHIP) was 0.77 ± 0.02 mm, 0.43 ± 0.03 mm, and 0.42 ± 0.02 mm (Figure 1(F)). At the time after the intervention, pinocembrin had improved the cardiac function similar to the SHAM group (*p* < .001). At the same times, we found that the concentration of NT-proBNP were significantly increased in the CIHF group compared with that of the SHAM group, which were attenuated by the treatment with pinocembrin (Figure 1(G), *p* < .001).

### 3.2. Effects of pinocembrin on ventricular electrical remodelling

**Figure 2.**
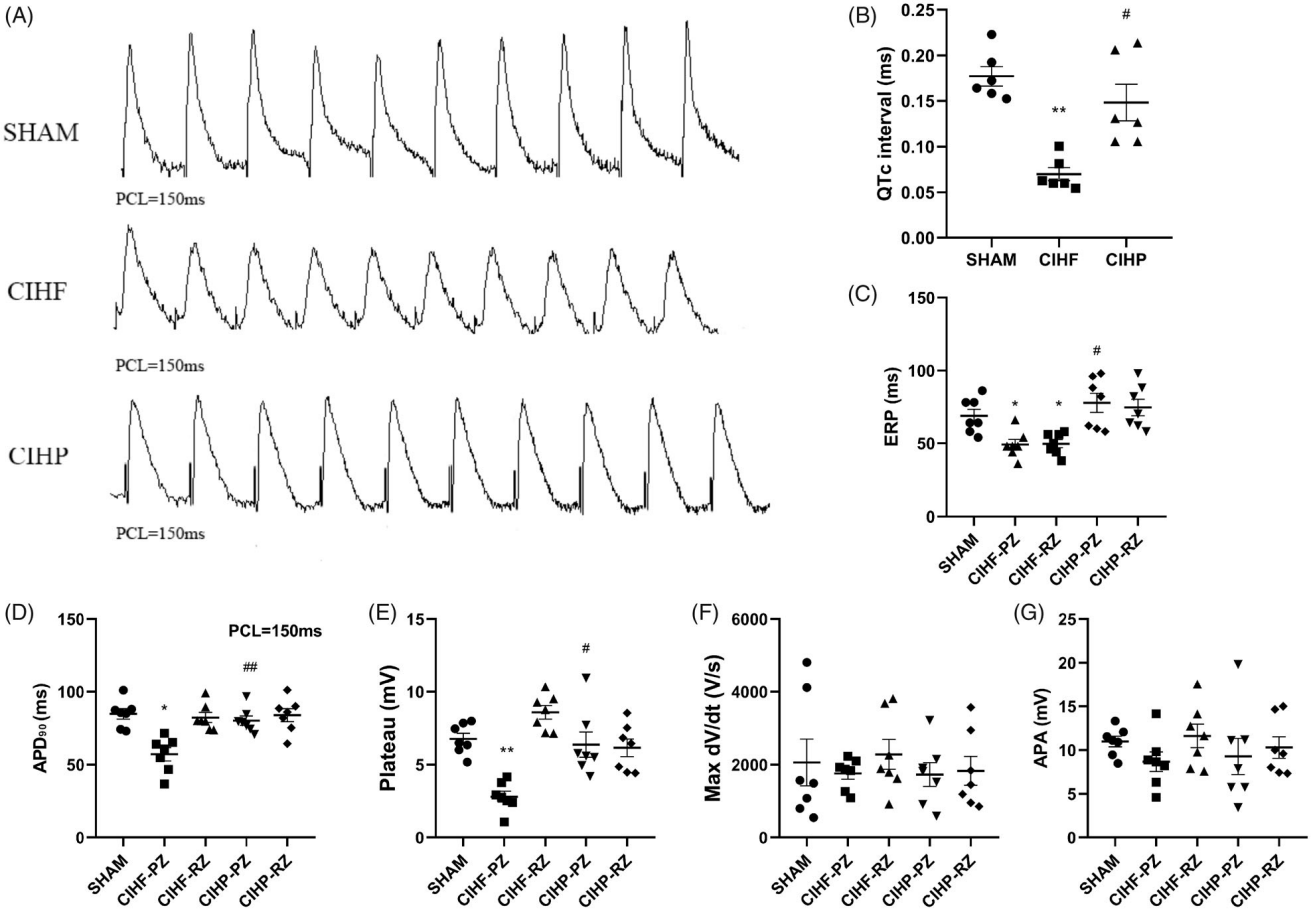
The effect of pinocembrin on the ECG and ventricular electrophysiological paraments (*n* = 7). (A) Representative recordings of APD in each group as PCL = 150 ms. (B–D) Correct QT interval, ERP and APD_90_ in each group, respectively. APD90 was defined as the duration between start and until when the action potential has repolarized by 90% of its height. (E–G) The plateau potential, APA and d*V*/d*t*_max_ in each group, respectively. Plateau potential is the average voltage measured in action potential plateau. Data are expressed as means ± SE. *p < .05 versus SHAM; #*p* < .05 versus CIHF; **p < .01 versus SHAM; ##*p* < .01 versus CIHF. APA: action potential peak voltage; Max d*V*/d*t*: the maximum of slopes of upstroke.

Ventricular electrical activity of HF underwent distinct changes (Figure 2(A)). After 1 month of intervention, the QTc interval was remarkably increased in CIHP compared with that of CIHF (SHAM: 0.17 ± 0.01 s; CIHF: 0.07 ± 0.007 s; CIHP: 0.14 ± 0.01 s; *p* < .05; Figure 2(B)). Compared with the SHAM group (68.86 ± 4.16 ms), the ERP in both CIHF-PZ (49.71 ± 2.60 ms) and CIHF-RZ (49.14 ± 3.23) was shortened (Figure 2(C)). Pinocembrin significantly prolonged ERP of CIHP (CIHP-PZ: 74.57 ± 5.25 ms; CIHP-RZ:77.71 ± 6.09 ms; *p* < .05).

Compared with the SHAM group (84.91 ± 3.29 ms), epicardium from the CIHF-PZ showed shortened APD_90_ (57.14 ± 4.20 ms), whereas CIHF-RZ (82.37 ± 3.18 ms) showed no changed. Treatment with pinocembrin prevented APD90 shortening at CIHP-PZ (80.24 ± 2.95 ms), whereas it did not affect the CIHP-RZ (83.97 ± 4.09 ms, Figure 2(D)).

Moreover, plateau potentials were decreased in the CIHF-PZ (2.81 ± 0.35 mV) and showed a tendency of prolongation in the CIHF-RZ (8.59 ± 0.43 mV). Meanwhile, pinocembrin exhibited positive effects on CIHP-PZ (6.39 ± 0.80 mV) and CIHP-RZ (6.17 ± 0.56 mV). However, there was no change in APA and the maximal upstroke velocity (d*V*/d*t*) among the zones of the SHAM, CIHF, and CIHP groups, respectively (Figure 2(E–G)).

**Figure 3.**
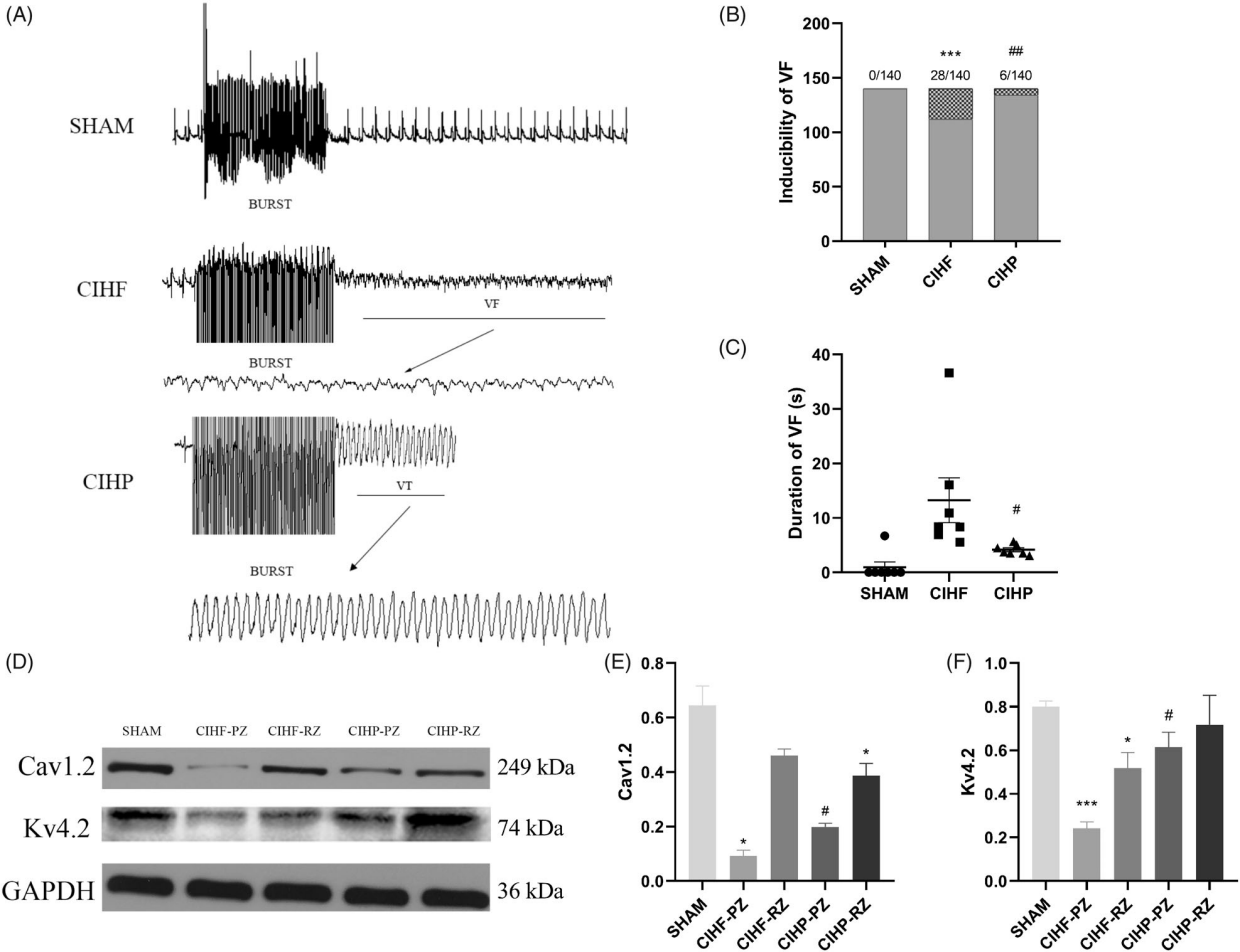
Effects of pinocembrin on vulnerability to ventricular fibrillation (*n* = 7) and cardiac ion channel protein (*n* = 3). (A) Representative recording of VF in each group. (B and C) Inducibility and duration of VFs, respectively. (D) Western blot analysis of Kv4.2 and Cav1.2. (E and F) Quantitative analysis of Cav1.2 and Kv4.2 expressed in the ventricle, respectively, and the histograms represent the ratio between protein and GADPH. Data are expressed as means ± SE. *p < .05 versus SHAM; #*p* < .05 versus CIHF; **p < .01 versus SHAM; ##*p* < .01 versus CIHF. ***p < .001 versus SHAM. PZ: peri-infarct zone; RZ: remote zone.

Figure 3(A) shows examples of VF induced using the burst pacing protocol. The susceptibility and average duration of VF in the CIHF group were markedly increased. Pinocembrin significantly suppressed ventricular vulnerability to fibrillation, as reflected by a significant decrease in average duration of VF and VF inducibility in the CIHP group (Figure 3(B,C), *p* < .05).

### 3.3. Pinocembrin decreases Cav1.2 and Kv4.2 expression

Western blots of CIHF-PZ samples revealed that Cav1.2 levels were lower compared with that of the SHAM, whereas the levels were increased following pinocembrin treatment. In addition, the levels of Cav1.2 in the CIHP-RZ were lower than that of the SHAM sample (Figure 3(E)). On the other hand, we found a significant decrease in Kv4.2 expressions in both the CIHF-PZ and CIHF-RZ groups compared with SHAM. After pinocembrin treatment, the Kv4.2 content in both the peripheral and remote borders increased. However, only the data of peripheral border had statistical significance (Figure 3(F), *p* < .05). These data suggested that complex and variable changes in the ionic currents may contribute to the VAs after CIHF.

### 3.3. Pinocembrin ameliorates sympathetic neural remodelling

**Figure 4.**
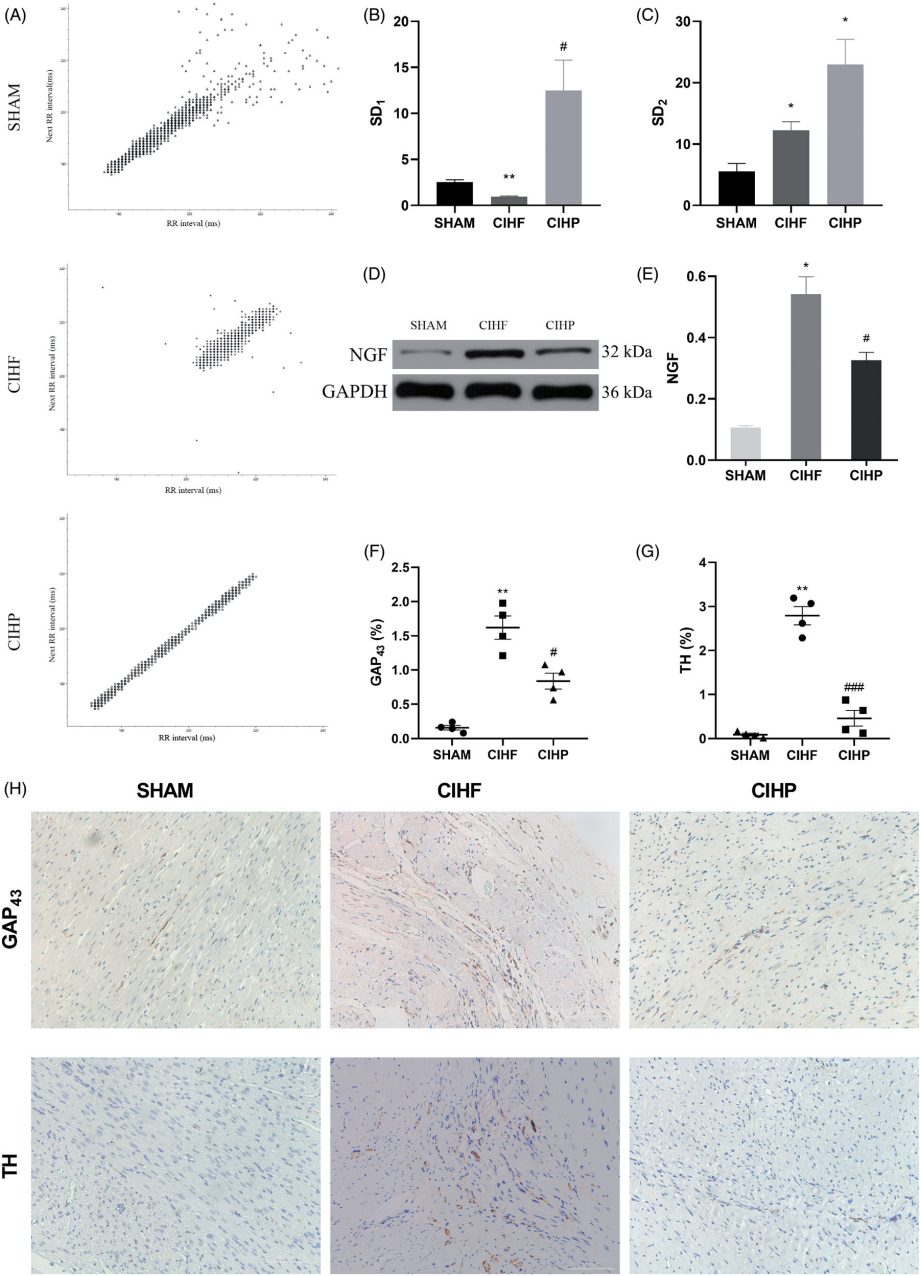
Effects of pinocembrin on autonomic nerve remodelling in CIHF (*n* = 3). Pinocembrin reduces the expression of sympathetic innervation related proteins and increases the parasympathetic activity. (A) The plot-Poincaré maps of each group. (B and C) The SD_1_ and SD_2_ in each group, respectively. SD_1_ indicates the parasympathetic activity, whereas the SD_2_ indicates a combination of sympathetic and parasympathetic activities. (D and E) The western blot and quantitative analysis of NGF protein expression. (F–H) The representative immunostaining and quantitative analysis of both TH and GAP43, respectively. Data are expressed as means ± SE. *p < .05 versus SHAM; #*p* < .05 versus CIHF; **p < .01 versus SHAM; ##*p* < .01 versus CIHF. ***p < .001 versus SHAM. ###*p* < .001 versus CIHF. NGF: nerve growth factor; GAP_43_: growth associated protein 43; TH: tyrosine hydroxylase.

We analyzed the parameters of heart rate variability (HRV), which represent autonomic nervous activity (Figure 4(A)). SD_1_, one of the Poincare plot map parameters, was decreased in the CIHF group (0.95 ± 0.06) compared with that of the SHAM group (2.52 ± 0.62, Figure 4(B)). The SD_1_ was higher following pinocembrin treatment (12.49 ± 3.01) in the CIHP group. Otherwise, SD_2_ (Figure 4(C)) was higher in both the CIHF and CIHP rats [14].

We studied the NGF expression of ventricles in each group (Figure 4(D)). Quantitative of the NGF protein increased in the CIHF group (0.55 ± 0.03) compared with that of the SHAM group (0.10 ± 0.003). Pinocembrin (0.32 ± 0.01) significantly curbed this trend, as reflected by expression of NGF protein in the CIHP group (Figure 4€).

We then observed that TH and GAP_43_ dispersed in the CIHF group and the CIHP group at 3 months post-MI, respectively (Figure 4(H)). The staining of both TH (2.79 ± 0.18%) and GAP_43_ (1.62 ± 0.15%) in the CIHF were increased compared with the SHAM group (TH:0.09 ± 0.03%; GAP_43_: 0.15 ± 0.03%). The CIHP group showed a significant decrease of TH (0.46 ± 0.15%) and GAP_43_ (0.84 ± 0.10%) nerve densities (Figure 4(F,G)).

### 3.4. Pinocembrin inhibits ventricle fibrosis and infarct area and mitigates HF-induced Cx43 reduction

**Figure 5.**
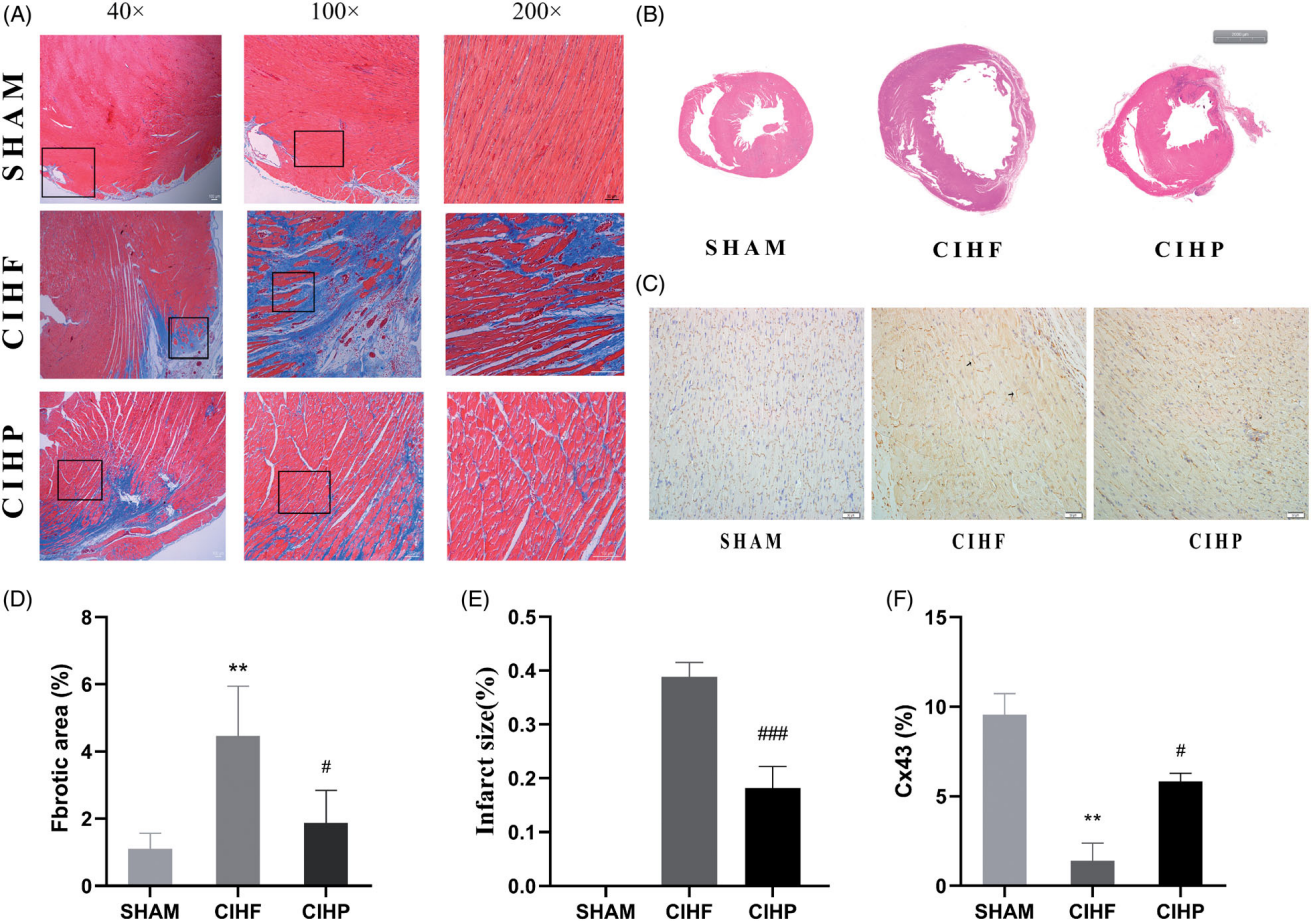
To assess the effect of pinocembrin on cardiac fibrosis, infarct area and Cx43(*n* = 3). (A) Representative Masson staining of LV in each group. (B) Representative HE staining of ventricles in each group. (C) Representative staining of Cx43. (D and E) Statistical diagram of cardiac fibrosis contents, infarct area and Cx43. → means abnormal Cx43 and →means normal Cx43. Data are expressed as means ± SE. #*p* < .05 versus CIHF; ###*p* < .001 versus CIHF; **p < .01 versus SHAM.C_X_43: connexin protein 43.

The extent of fibrosis (6.69 ± 1.89% versus 1.10 ± 0.17%, Figure 5(A,D)) and infarct area (0.38 ± 0.02% versus 0, Figure 5(B,E)) increased in the CIHF group compared with that of the SHAM group, whereas that were significantly attenuated in the CIHP group (1.87 ± 0.36% and 0.18 ± 0.03, respectively).

The results of immunohistochemistry showed (Figure 5(C,D)) that the expression of C_X_43 was significantly decreased in the CIHF group (1.39 ± 0.33) comparing with that of the SHAM group (9.56 ± 0.39). The parameter was significantly increased in the ventricle of CIHP (5.83 ± 0.15) due to pinocembrin intake.

## 4. Discussion

**Figure 6.**
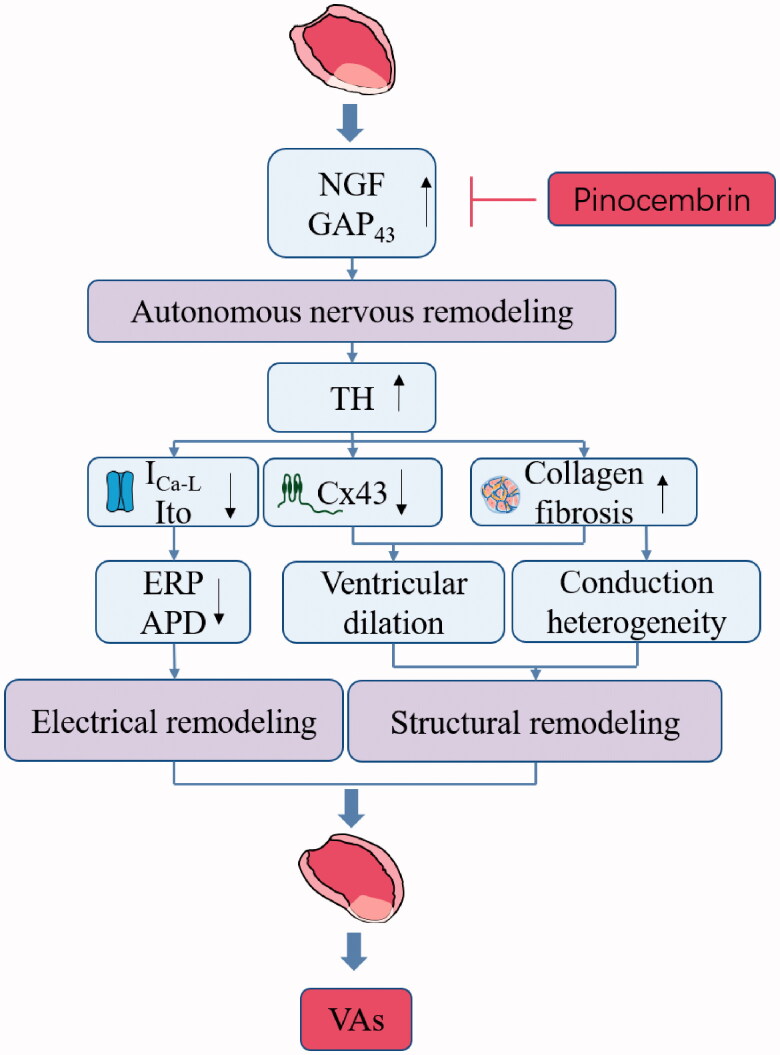
Summary of schematic showing of the reversal effect of pinocembrin on VAs.

Here we studied the efficacy of pinocembrin on susceptibility to Vas in an animal model of CIHF to understand its potential mechanism. Pinocembrin treatment decreased the VFs susceptibility and average duration of VFs in the CIHP rat. This was followed by the prolongation of ERP at both the peri-infarct and remote zones following pinocembrin treatment. Furthermore, pinocembrin inhibited the shortening of APD in peri-infarct zone of CIHF. Additionally, treatment with pinocembrin led to the upregulation of Cav1.2 and Kv4.2. Simultaneously, pinocembrin decreased cardiac autonomic remodelling. Pinocembrin notably downregulated cardiac NGF, TH and GAP_43_. In CIHP rats, the collagen fibres and infarct area decreased, whereas C_X_43 increased. These findings suggested that pinocembrin acted on autonomic remodelling to blocking the susceptibility to VAs after CIHF (Figure 6).

### 4.1. Pinocembrin and VAs

Recent findings showed that one of the components from propolis, pinocembrin, contributes to anti-inflammatory, immune-boosting, anti-oxidant, neuroprotective, and cardioprotective effects [15]. Our previous study demonstrated that pinocembrin decreased MI-induced atrial fibrillation susceptibility [13]. In the present study, CIHF rats had poor cardiac function compared with the SHAM group. Following pinocembrin treatment, rats in the CIHF group showed remarkable improvement. A series of studies determined the value of decreased RWT of left ventricle in predicting risk of Vas [16]. Interestingly, we have found that pinocembrin increased RWT in CIHF rats, which provided a preliminary indication of its antiarrhythmic effects.

Currently, CIHF has been associated with common arrhythmic disorders, such as atrial fibrillation, and more fatal processes, including VAs and SCD [17]. VAs are a common complication of patients with CIHF. It is demonstrated that VAs are associated with the heterogeneity of APD and ERP [18]. The APD is primarily responsible for the repolarization of the heart. The extensive structural and functional remodelling in CIHF leads to the dispersion of APD, which causes VAs. Our study showed the similar results. Compared with the SHAM group, the decreased ERP and APD and increased VF susceptibility were observed in the CIHF group. Pinocembrin significantly increased the mean ERP and APD in the peri-infarct zone and reduced the heterogeneity of cardiac repolarization and inducibility rate of VF.

Various ion currents affect the plateau phase, which provides a substrate for VAs. The plateau phase is a period of high membrane resistance, and during which small changes in current can easily upset the balance. Ito has a significant effect on the level of plateau and subsequently on all of the currents, which are activated later in the APD [19]. The functional decline of the transient outward K^+^ (Ito) is a recurring theme in failing ventricular myocardium. Moreover, Ito is regulated by neurohumoral mechanisms, and the stimulation of α-adrenergic receptors significantly reduces the current amplitude of Ito. The changes in I_Ca-L_ also contribute to electrical instability. In human CIHF, intracellular Ca^2+^and the APD are intricately bound through I_Ca-L_, a reduction expression and increment phosphorylation of Cav1.2 both leading to positive Ca^2+^-APD coupling [20]. Abnormal I_Ca-L_ cause calcium oscillations and DADs mediated VAs. Our results showed that the expression of Cav.1.2 and Kv4.2 was markedly increased after the treatment with pinocembrin.

The structure changes characterized by excessive replacement of extracellular matrix proteins in the myocardium, which is the basis of arrhythmias [21]. There is a significant fibrosis deposition in the CIHF group. Fibrosis causes inhomogeneous and anisotropic conduction, leading to re-entry arrhythmia and interruption of conduction waves. In addition, Cx43 is the major kind of connexin in ventricles, and a slowed conduction velocity and fatal arrhythmias align with decreases in Cx43 expression [22]. Our results showed that the decreased C_X_43and increased fibrosis extent were observed in the CIHF group comparing with that of the SHAM group.

### 4.2. The autonomic remodelling and VAs

Autonomic nervous system (ANS) is a crucial role in the pathogenesis of VAs in CIHF. On the one hand, increased sympathetic activation can lead to increased automaticity of the ventricular pacemaker cells. On the other hand, ANS also regulates early afterdepolarization (EADs) and delayed afterdepolarization (DADs) [23]. Previous studies have shown that the density of sympathetic nervous in myocardial infarction area of spontaneous VAs patients was higher than that of non-spontaneous VAs patients [24]. It is reported that the density of nervous in left stellate ganglion increased significantly after myocardial infarction caused by coronary artery balloon occlusion [25]. Moreover, sympathetic activation reduces ERP and QTc, which could be a prerequisite for circus-type re-entry [26]. Furthermore, sympathetic neurotransmitters increase excitation and conduction heterogeneity and lead to susceptibility to VAs by interacting with cardiac ion channels. Sustained sympathetic activation inhibits Kv4.3, depolarizes L-type calcium channels, and resulting in APD shortening [27, 28]. Our electrophysiological data support these published findings. At the molecular level, autonomic nerves not only acted on ion channels, but also interacted with connexin proteins extensively. Yang et al. found that the neural chemorepellent semaphoring 3a inhibits neural remodelling, reducing the accumulation of dephosphorylated Cx43, and improving the inductivity of VAs [29]. These findings are consistent with our observation that up-regulated Cx43 were parallel with the reduced incidence of VTs following pinocembrin treatment.

### 4.3. Pinocembrin and autonomic remodelling

During the progression of CIHF, NGF and GAP_43_ are upregulated at the infarcted area. The NGF and GAP_43_then result in the sprouting of nerves at the non-infarcted and infarcted area [30]. At the cardiac ventricle, sympathetic effect is prepotent. Regional sympathetic activation and hyperinnervation cause mechanical and electrical heterogeneity, and increase susceptibility to Vas [31]. We showed that pinocembrin treatment inhibited the expression of NGF in the ventricle, followed by the reduction of GAP_43_ and TH. Our previous study showed that the pinocembrin significantly decreased cardiac autonomic remodelling and improved HRV in the myocardial ischaemia rats. Interestingly, our results are consistent with the studies. The increasedSD_1_of plot-Poincaré maps indicates an increase in parasympathetic activity, whereas the SD2 index indicates a combination of sympathetic and parasympathetic activities [32]. Based on our data, pinocembrin increased parasympathetic activity and alleviated neural remodelling.

## 5. Limitation

There are some limitations in this study. First, there are many ion channels that affect the plateau phase. We pay more attention to Ito and I_Ca-L_. However, although the Ito and I_Ca-L_ are dominant currents in the repolarization period for both humans and rats, the effect of Ito in rats is significantly greater than that in humans. In addition, the delayed rectifier K current and the I_Na/Ca_ are both the important repolarization currents that affect the human ventricular action potential, which may not be obvious in rat ventricular myocytes. The further studies required. Second, inflammatory responses and oxidative stress can also lead to adverse cardiac outcomes, which needs more subsequent studies. Finally, we focus on the inhibitory effect of pinocembrin on VAs, and its effect on the SHAM group may requires further evaluation. Nevertheless, we still provide a potential target for the treatment of CIHF and VAs.

## 6. Conclusion

In the current study, pinocembrin can attenuate cardiac autonomic nerve remodelling and ventricular fibrosis of CIHF rat models, and thus improve ion channel remodelling, ventricular electrical heterogeneity, and VFs susceptibility. The results showed that pinocembrin may be a promising drug for patients with CIHF.

## Data Availability

The data that support the findings of this study are available from the corresponding author upon reasonable request.

## Abbreviations

ANS: autonomic nervous system
APD: action potential duration
CIHF: chronic ischaemic heart failure
ERP: effective refractory period
I/R: ischaemia/reperfusion
MI: myocardial infarction
SCD: sudden cardiac death
VAs: ventricular arrhythmias
VFs: ventricular fibrillations
VTs: ventricular tachyarrhythmias
